# Combined seed and foliar pre-treatments with exogenous methyl jasmonate and salicylic acid mitigate drought-induced stress in maize

**DOI:** 10.1371/journal.pone.0232269

**Published:** 2020-05-01

**Authors:** Nimrah Tayyab, Rabia Naz, Humaira Yasmin, Asia Nosheen, Rumana Keyani, Muhammad Sajjad, Muhammad Nadeem Hassan, Thomas H. Roberts

**Affiliations:** 1 Department of Biosciences, COMSATS University Islamabad, Islamabad, Pakistan; 2 Plant Breeding Institute, Sydney Institute of Agriculture, University of Sydney, Sydney, Australia; University of Agriculture, PAKISTAN

## Abstract

Susceptibility of plants to abiotic stresses, including extreme temperatures, salinity and drought, poses an increasing threat to crop productivity worldwide. Here the drought-induced response of maize was modulated by applications of methyl jasmonate (MeJA) and salicylic acid (SA) to seeds prior to sowing and to leaves prior to stress treatment. Pot experiments were conducted to ascertain the effects of exogenous applications of these hormones on maize growth, physiology and biochemistry under drought stress and well-watered (control) conditions. Maize plants were subjected to single as well as combined pre-treatments of MeJA and SA. Drought stress severely affected maize morphology and reduced relative water content, above and below-ground biomass, rates of photosynthesis, and protein content. The prolonged water deficit also led to increased relative membrane permeability and oxidative stress induced by the production of malondialdehyde (from lipid peroxidation), lipoxygenase activity (LOX) and the production of H_2_O_2_. The single applications of MeJA and SA were not found to be effective in maize for drought tolerance while the combined pre-treatments with exogenous MeJA+SA mitigated the adverse effects of drought-induced oxidative stress, as reflected in lower levels of lipid peroxidation, LOX activity and H_2_O_2_. The same pre-treatment also maintained adequate water status of the plants under drought stress by increasing osmolytes including proline, total carbohydrate content and total soluble sugars. Furthermore, exogenous applications of MeJA+SA approximately doubled the activities of the antioxidant enzymes catalase, peroxidase and superoxide dismutase. Pre-treatment with MeJA alone gave the highest increase in drought-induced production of endogenous abscisic acid (ABA). Pre-treatment with MeJA+SA partially prevented drought-induced oxidative stress by modulating levels of osmolytes and endogenous ABA, as well as the activities of antioxidant enzymes. Taken together, the results show that seed and foliar pre-treatments with exogenous MeJA and/or SA can have positive effects on the responses of maize seedlings to drought.

## Introduction

Maize is a highly productive, multi-use food or forage cereal crop of critical importance globally. In Pakistan, a country where the human population is continually increasing, maize is the third most important crop (after wheat and rice), representing about 4.8% of the total agricultural area and 3.5% of the value of agricultural output [1].

Water scarcity is the major environmental limitation worldwide to plant growth, development and yield [2]. It has a range of influences on plants, including reduced membrane integrity, osmotic effects, pigment content, plant growth and photosynthetic activity [3]. While maize productivity is high in many regions of the world, in some countries (e.g. Pakistan) it is limited because of plant exposure to severe biotic and abiotic stresses, including drought [4]. Oxidative stress is often thought of as a secondary stress of drought stress. The damage to biological macromolecules caused by reactive oxygen species (ROS) under drought stress are among the key limitations to plant growth [5]. Inadequate availability of soil water diminishes maize metabolic activity, reduces biomass, and lowers photosynthetic rate by decreasing the leaf chlorophyll content, ultimately leading to decreased maize yield [6,7]. Drought stress during the early growth stages of maize can seriously inhibit its leaf area, growth and yield [8,9].

Plant hormones play a crucial role as signaling molecules in developmental processes, defense responses and responses to abiotic stress [10,11]. Exogenous applications of specific growth regulators can increase the tolerance of plants to various environmental stresses [12,13,14,15]. Methyl jasmonate (MeJA), a naturally occurring plant growth regulator, affects morpho-physiological and biochemical plant processes including seed germination, root growth, fruit ripening, and senescence [16]. The exogenous application of MeJA can help provide tolerance to abiotic stresses including drought and salinity, and in defense-oriented metabolism of plants [17,18,19]. Jasmonic acids (JA) play a significant role in signaling of water stress-induced antioxidant responses, via ascorbate metabolism [3, 20]. The exogenous application of JA has been found effective in providing protection against drought-induced oxidative stress in muskmelon (*Cucumis melo*) by enhancing the activity of antioxidant enzymes [21]. In *Brassica napus* under metal stress, MeJA application increases the scavenging of reactive oxygen species (ROS) by enhancing the levels of secondary metabolites, stimulates the antioxidant defense system and decreases arsenic content [22]. Soaking of maize seeds in MeJA can alleviate the harmful effects of drought stress by increasing total protein, proline, carbohydrate content and antioxidant activities [23].

Salicylic acid (SA) is another growth regulator known to maintain growth and development of plants and responses to several biotic and abiotic stresses [24,25]. SA upregulates genes that encode chaperones, antioxidant enzymes, heat shock proteins and several other gene products involved in metabolizing secondary metabolites [25]. It provides protection to plants against abiotic stresses by the regulation of important physiological processes including photosynthesis, nitrogen and proline metabolism, the antioxidant defense system and responses of plants to water deficit [24,25,26,27,28,]. Water deficit decreases stomatal conductance and photosynthetic rate, which results in reduced plant growth [29]. Exogenous applications of SA to leaves under drought stress increases stomatal conductance, which leads to increases in plant dry mass in barley [30]. Under water deficit, the exogenous application of SA increases the rates of specific enzymatic reactions that decrease the level of oxidative stress [31]. SA application increases the antioxidant defense system under water deficit [32], which ultimately increases plant biomass [33]. In rice under quinclorac stress, exogenous SA applications regulated the antioxidant defense system, protected cell organelles from degradation and reduced ROS formation [34]. In maize under salt stress, exogenous seed treatment with SA (2 mM) increased seedling emergence and establishment [35].

The objective of this study was to determine the effects of exogenous application of MeJA and SA, alone and in combination as pre-treatments to seeds and leaves, on various morpho-physiological and biochemical properties of maize seedlings under drought stress and well-watered conditions. The interactive effects of combined MeJA and SA applications on the maize detoxification system under drought stress have not been reported. The present study provides a new insight into MeJA+SA-induced coordinated effects on antioxidant defense and the endogenous phytohormone system to enhance resistance against drought-induced oxidative stress in maize seedlings. Parameters investigated included levels of osmolytes, activities of antioxidant enzymes and levels of endogenous abscisic acid (ABA).

## Materials and methods

Seeds of maize cv. CM451 NARC were obtained from the National Agricultural Research Centre (NARC), Islamabad, Pakistan. Maize seeds were sterilized with 95% (v/v) ethanol and subsequently 10% (v/v) Clorox for 2–3 min and rinsed thoroughly 3X with sterile distilled water.

### Experimental setup

Seeds were soaked in solutions of methyl jasmonate (MeJA; 20 μM) and/or salicylic acid (SA; 2 mM) for 18 h prior to sowing, while seeds for the combined applications were soaked in 10 μM MeJA + 1 mM SA. Control plants were soaked in sterile water. Seeds were divided into two groups, drought treated and control, and sown in pots (18 cm diameter × 23 cm height) containing equal amounts of sandy loam soil (sand 84%, silt 13%, clay 3%). Seven seeds were sown per pot and were thinned to five plants one week after planting. Two weeks after sowing, maize seedlings were again treated with MeJA and/or SA solutions as foliar applications 24 h before drought stress. This was done to further enhance the possible effects of these growth regulators on reducing the impact of drought stress on the maize seedlings. Water deficit was imposed for 5 d on the drought-treated plants while the controls remained well-watered. Three pot replicates were used for each treatment and were arranged in a completely randomized design (CRD) in a growth room, with the average temperature of 25–30°C and with photoperiod conditions of 13/11 h (light/dark).

### Growth parameters

At the time of harvesting (45 d from sowing and following 5 d of drought stress), root length (RL), shoot length (SL), root dry weight (RDW) and shoot dry weight (SDW) were measured (three replicates from each treatment). RDW and SDW were determined using a microwave oven on medium power for 1.5 min ± 30 s until constant weight [36].

### Physiological and biochemical parameters

#### Soil moisture content (SMC)

Soil moisture content was determined according to Reeb and Milota [37]. Soil (20 g) was taken at a uniform depth of 6 cm from the surface. Fresh weight of the samples (three replicates from each treatment) was recorded. Dry weight was determined after drying the soil in an oven for 72 h at 70°C until constant weight.

#### Relative water content (RWC)

The RWC of leaves (three replicates from each treatment) was determined following the method of Gupta [38] with some modifications and calculated using the formula below:

RWC(%)=[(FW-DW)÷(FTW-DW)]×100

where

FW=freshweight,DW=dryweight,FTW=fullyturgidweight

#### Chlorophyll and carotenoid contents

Chlorophyll and carotenoid extraction were performed according to the method of Hiscox and Tsraelstam [39] with some modifications. Leaf samples (0.1 g) (three replicates from each treatment) were soaked individually in 5 mL dimethyl sulfoxide (DMSO). A set of test tubes were incubated in a water bath at 60°C for 30 min. The absorbance of the samples was measured using a spectrophotometer at A_663_, A_645_ and A_470_ nm for chlorophyll *a*, chlorophyll *b* and carotenoid content, and the following equations applied:

Chla(mg/g)=12.7×A663-2.69×A645

Chlb(mg/g)=22.9×A645-4.68×A663

Carotenoidcontent(mg/g)=(4×A470nm×volumeofsample)/Freshweightofsample

#### Total soluble protein content

Leaf protein content was determined following the method of Lowry et al. [40]. The absorbance of each sample (three replicates from each treatment) was recorded at A_650_ nm after incubation for 30 min. The concentration of soluble protein content was determined with reference to a standard curve using bovine serum albumin (BSA).

#### Relative membrane permeability (RMP)

Flag leaves (three replicates from each treatment) were cut into equal pieces and transferred to test tubes containing 20 mL of deionized distilled water. The suspensions were vortexed for 10 s and the solution was assayed for initial electrical conductivity (EC0). The suspensions were then kept at 4°C for 24 h and then assayed for EC1. The same samples were autoclaved at 121°C for 20 min to determine EC2. Percent RMP was calculated following the formula described by Yang et al. [41]:

RMP(%)=EC1−EC0/EC2−EC0×100

#### Total soluble sugars (TSS)

TSS was determined based on the phenol-sulfuric acid method [42]. Leaf tissue (0.5 g) (three replicates from each treatment) was homogenized with deionized water, and the extract was filtered and treated with 5% phenol and 98% sulfuric acid. The reaction mixture was incubated for 1 h and the absorbance read at A_485_ nm.

#### Total carbohydrate content (TCC)

Determination of total carbohydrates was carried out according to the method described by Herbert et al. [43]. Dried maize leaf tissue (0.2–0.5 g) (three replicates from each treatment) was placed in a test tube, and sulfuric acid (1N; 10 mL) added to the sample. The sealed tubes were then placed in an oven overnight at 100°C. After filtration the solution was transferred to a flask (100 mL) and made up to 100 mL with distilled water. An aliquot (1 mL) of this solution was transferred to a test tube and treated with aqueous phenol solution (1 mL of 5%) followed by concentrated sulfuric acid (5 mL). After 10 min of shaking, the tubes were placed in a water bath at 23–30°C for 20 min. The absorbance of the solution was measured at 490 nm.

#### Proline content

Leaf sample (0.1 g) (three replicates from each treatment) was ground in 5 mL of 80% ethanol and centrifuged at 2500 rpm for 1 min at 4°C. In a separate flask, a reaction mixture was prepared containing ninhydrin 1% (w/v) in acetic acid 60% (v/v) and ethanol 20% (v/v) [44]. Supernatant (500 μL) was taken from each sample and 1 ml of reaction mixture was added. The absorbance was measured at 520 nm.

#### Oxidative stress indicators

*Lipid peroxidation*. Lipid peroxidation was determined as the amount of malondialdehyde (MDA) produced using a thiobarbituric acid (TBA) reaction according to Zhang [45] with some modifications. Leaf samples (1 g) from each treatment/replication (three replicates from each treatment) were homogenized with 1% trichloroacetic acid (TCA; 5 mL) and centrifuged at 10,000 *g* for 20 min. The supernatant (1 mL) was added to TCA (1 mL; 20%) containing 0.5% TBA. The reaction mixture was heated at 90°C for 35 min. The absorbance was measured at 532 and 600 nm. Lipid peroxidation was expressed as MDA content in nmol g^-1^ FW.

*Hydrogen peroxide (H*_*2*_*O*_*2*_*)*. Endogenous H_2_O_2_ content was measured according to the method of Velikova et al. [46]. Leaves (0.25 g) (three replicates from each treatment) were ground in 5% TCA (3 mL) with activated charcoal (0.1 g) at 0°C. The homogenate was centrifuged at 12,000 *g* for 15 min. Potassium phosphate buffer (0.5 mL; 10 mM, pH 7) and potassium iodide (0.7 mL; 1 M) were added to the supernatant (0.5 mL). The absorbance of each sample was measured at 390 nm, and H_2_O_2_ content was expressed as μmol g^−1^ dry weight.

#### Endogenous ABA

Leaf tissues (0.5 g) from each replicate and treatment (three replicates from each treatment) were homogenized with absolute methanol (3 mL) and kept at 4°C overnight. The extracted samples were filtered with sterile syringe filter (0.22 μm), concentrated by evaporation [47], and dissolved in HPLC-grade methanol. The filtrate (20 μL) was applied to HPLC (Agilent 1100, Germany) for the determination of endogenous ABA at 220 nm. Phytohormone (ABA) was eluted at 1 mL/min flow rate with gradient of methanol:acidic water (deionized water containing 0.67% acetic acid, pH 3.0) [48]. The system was equipped with a C18 column (39 × 300 mm) and a U.V. detector.

#### Enzyme extraction for antioxidant activities

Plant samples (three replicates from each treatment) for catalase (CAT), peroxidase (POD) and superoxide dismutase (SOD) assays were extracted by homogenizing leaf samples in 0.1 M phosphate buffer (pH 7.5) containing 1% polyvinylpyrrolidine (PVP), 1 mM EDTA and 10 mM β-mercaptoethanol. The homogenates were centrifuged at 10,000 *g* for 20 min and the supernatant was used for the assays (as described below).

*Catalase activity*. Catalase activity was performed according to the method of Kumar et al. [49] with some modifications. Supernatant (50 μL) was mixed with 3.4 mL phosphate buffer (25 mM, pH 7.0, containing 0.1 mM EDTA) and 200 μl H_2_O_2_. Enzyme activity was determined by measuring the change in absorbance at 240 nm after 1 min.

*Peroxidase activity*. Peroxidase activity was performed using the method of Vetter et al. [50] as modified by Gorin and Hidema [51] with some further modifications. An aliquot (50 μL) of sample (three replicates from each treatment) was mixed with 0.675 mL of 100 mM MES buffer (pH 5.5), 100 μL of 0.1% phenylenediamine and 0.3 μL of 0.05% H_2_O_2_. Absorbance was recorded at 485 nm for 3 min.

*Superoxide dismutase activity*. SOD activity was assayed by the method of Beauchamp and Frodovich [52]. The reaction mixture contained 1.17 μM riboflavin, 0.1 M methionine, 56 μM nitro blue tetrazolium chloride (NBT) and 0.05 M phosphate buffer. Two sets of reaction mixtures were prepared: one was kept in the light at 30°C and the other in the dark for 20 min. The absorbance was measured at 560 nm.

### Statistical analysis

All data were subjected to analysis of variance (ANOVA). Mean differences (three replicates from each treatment) were compared by an LSD test [53] using Statistix 8.1. Differences at p ≤0.05 were considered significant. To elucidate the effects of exogenous applications of MeJA/SA on responses of maize seedings under control and drought stress, a Pearson correlation analysis was performed using Statistix 8.1. A heatmap and cluster analysis was performed using ClustVis (www.biit.cs.ut.ee/clustvis/). A principal component analysis (PCA) was performed using Primer 6.

## Results

### Effects of MeJA and SA on Soil Moisture Content (SMC) and leaf Relative Water Content (RWC) under drought stress

**Fig 1:**
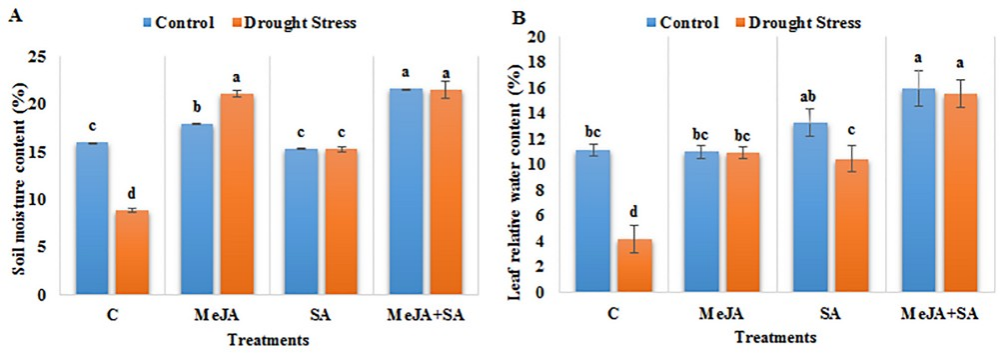
Effect of exogenously applied methyl jasmonate (MeJA) and salicylic acid (SA) on (A) soil moisture content and (B) leaf relative water content of maize seedlings grown under control (well-watered) and drought stress conditions. The data represent means of three replicates and error bars show ± standard errors of the mean values. Mean values that share the same letter are not-significantly different; otherwise they differ at p<0.05. C = control, MeJA = 20 μM methyl jasmonate, SA = 2 mM salicylic acid, MeJA+SA = 10 μM methyl jasmonate + 1 mM salicylic acid.

After 5 d of water deficit, visible effects including folding and wilting of leaves of the maize plants were observed, as expected. Soil moisture content (SMC) was significantly lower for the drought-stressed plants than in the control (9% vs 16%), verifying that these symptoms were indicative of water deficit. The exogenous applications of MeJA applied alone as well as in combination with SA increased the SMC under control and drought conditions, respectively, compared to their corresponding controls (Fig 1A). The maximum increase (142%) in SMC was observed for the combined application of MeJA+SA under drought stress.

Relative water content (RWC) of maize leaves was decreased by 62% under drought stress compared to the well-watered plants. The exogenous applications of MeJA and SA increased the RWC irrespective of stress. The maximum increase was exhibited with application of MeJA+SA by 43% and 272% under unstressed and drought stress conditions, respectively, compared to their controls (Fig 1B).

### Effects of MeJA and SA on plant growth parameters under drought stress

**Fig 2:**
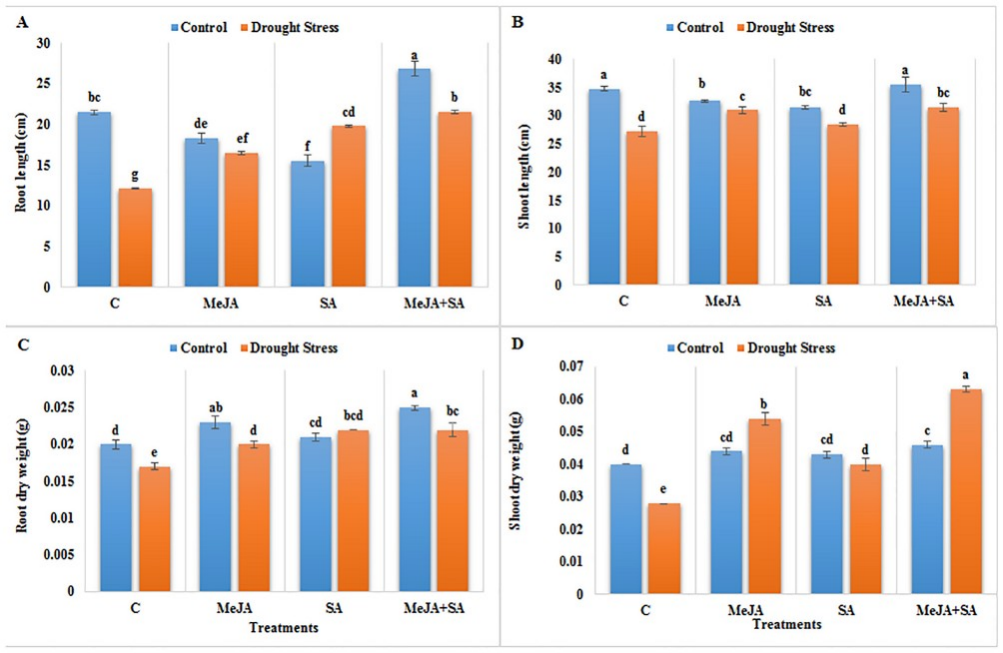
Effect of exogenously applied MeJA and SA on (A) root length (B) shoot length (C) root dry weight and (D) shoot dry weight of maize seedlings grown under control (well-watered) and drought stress conditions. The data represent means of three replicates and error bars showing ± standard errors of the mean values. Mean values that share the same letter are not-significantly different; otherwise they differ at p<0.05.

Root and shoot lengths of the maize plants were significantly decreased under drought stress (Fig 2A and 2B). Drought stress-plants exhibited lower shoot and root length as compared to the non-stressed control. Pre-treatment with MeJA and SA when applied alone did not significantly affect the root and shoot lengths, but their combined application significantly increased the lengths of roots under unstressed conditions compared to the well-watered plants that were not pre-treated. All the hormone pre-treatments of drought-stressed plants exhibited higher shoot and root lengths compared to stressed plants without hormone pre-treatment. The maximum increases in shoot and root lengths were observed for the combined application of MeJA+SA under drought stress conditions, which were 15 and 77%, respectively, at p<0.05.

Exogenous application of MeJA and SA alone and in combination increased the root dry weight (RDW) and shoot dry weight (SDW) significantly both under stressed and well-watered conditions (Fig 2B and 2C). Drought stress significantly decreased the RDW and SDW by 15% and 30%, respectively, compared to the well-watered control. Single and combined applications of MeJA and SA increased the RDW and SDW under drought stress when compared to drought-stressed plants not treated with hormones. Increases in RDW and SDW were most pronounced, at 29% and 125%, respectively, for the combined application of MeJA+SA.

### Effects of MeJA and SA on leaf chlorophyll, carotenoid and protein contents under drought stress

**Fig 3:**
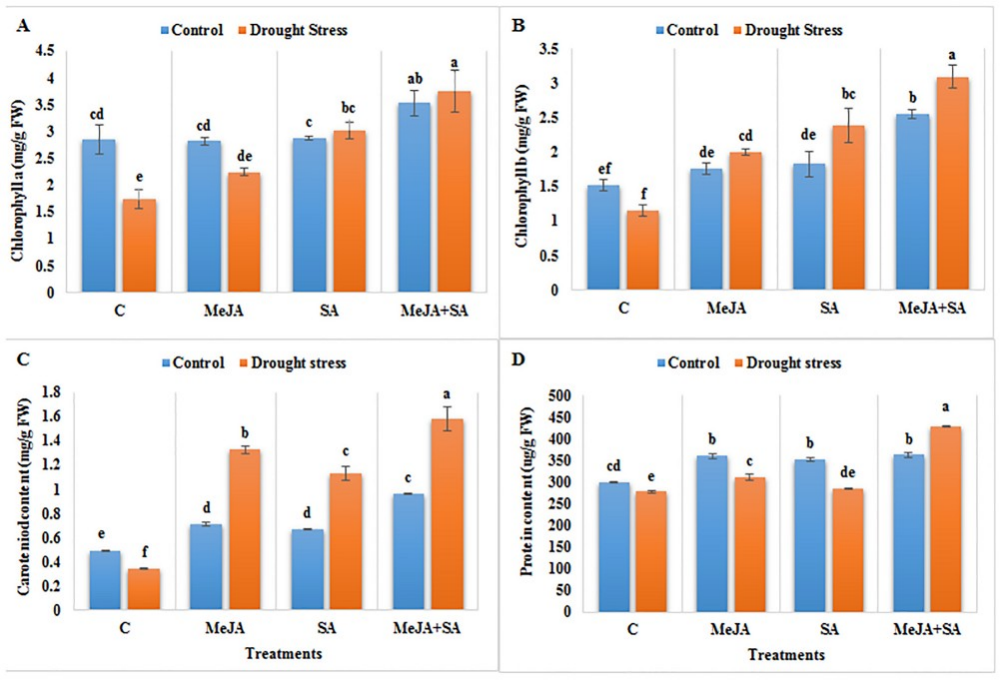
Effect of exogenously applied MeJA and SA on (A) chlorophyll *a* (B) chlorophyll *b* (C) carotenoid content and (D) protein content of maize seedlings grown under control (well-watered) and drought stress conditions. The data represent means of three replicates and error bars showing ± standard errors of the mean values. Mean values that share the same letter are not-significantly different; otherwise they differ at p<0.05.

Drought stress decreased the levels of leaf chlorophyll *a*, chlorophyll *b* and carotenoids by 64, 32 and 43%, respectively, compared to the well-watered plants (Fig 3A, 3B and 3C). The exogenous applications of MeJA and SA, alone and particularly in combination, reduced these effects of drought stress. MeJA+SA application for the drought-stressed plants resulted in levels of chlorophyll *a*, chlorophyll *b* and carotenoid contents that were 116, 167 and 358% higher, respectively, compared to the drought-stressed plants without hormone pre-treatment.

Drought stress was associated with a 7% decrease in protein content as compared to the non-stressed control plants. Exogenous application of MeJA and SA lead to a higher protein content under well-watered as well as drought stress conditions compared to their respective control plants. MeJA+SA treatment under drought stress exhibited the highest increase in protein content (54%) compared to the drought-stressed plants without pre-treatment with hormones (Fig 3D).

### Effects of MeJA and SA on relative membrane permeability and osmolytes under drought stress

**Fig 4:**
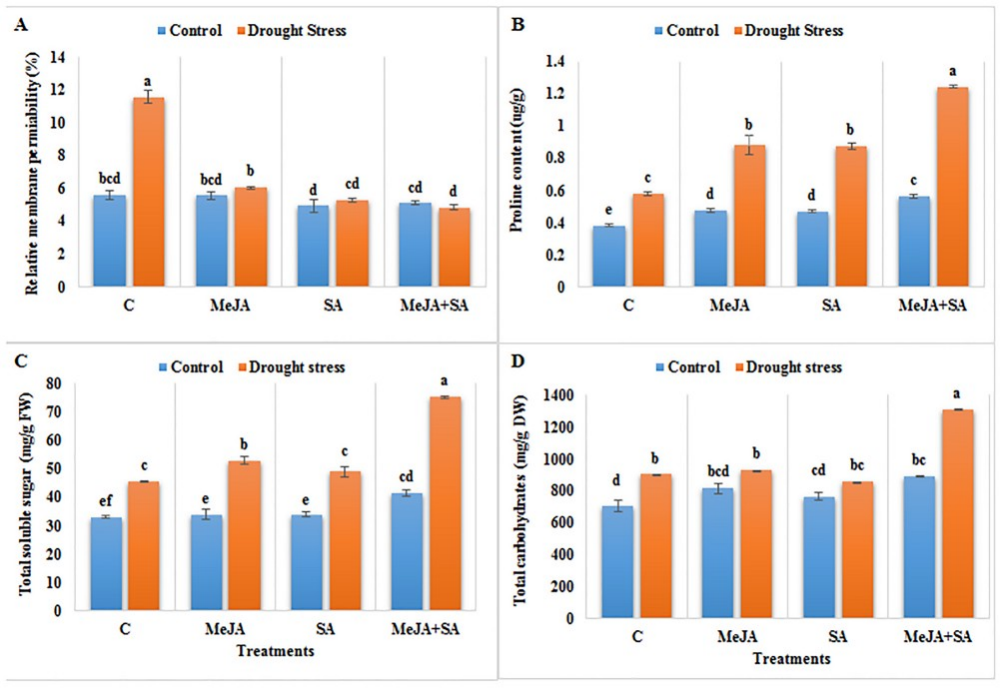
Effect of exogenously applied MeJA and SA on (A) relative membrane permeability (B) proline content (C) total soluble sugar and (D) total carbohydrate content of maize seedlings grown under control (well-watered) and drought stress conditions. The data represent means of three replicates and error bars showing ± standard errors of the mean values. Mean values that share the same letter are not-significantly different; otherwise they differ at p<0.05.

Relative membrane permeability (RMP) of maize plant leaves was increased by 106% under drought stress compared to the well-watered plants (Fig 4A). Exogenously applied MeJA and SA ameliorated this effect. The protective effect of MeJA+SA under drought stress was the most pronounced, decreasing RMP by 58% compared to drought-stressed plants not pre-treated with exogenous hormones.

Drought stress increased the proline content by 51% compared to the well-watered controls (Fig 4B). However, the exogenous applications of MeJA and SA prior to drought stress gave an even higher level of proline. The greatest effect was observed in the MeJA+SA treated seedlings, which had a proline content that was 115% higher under drought-stressed conditions compared to stressed plants without pre-treatment with exogenous hormones.

Increases in the total soluble sugar (TSS) and total carbohydrate content (TCC) by 38 and 28%, respectively, were observed in drought-stressed plants compared to the well-watered controls (Fig 4C and 4D). Exogenously applied MeJA significantly increased TSS by 16% while SA was found to be ineffective in increasing TSS compared to the stressed plants without pre-treatment. While MeJA and SA when applied alone were not effective in increasing TCC under drought stress, their combined application resulted in increases in TSS and TCC by 65 and 45%, respectively, compared to drought-stressed plants not pre-treated with exogenous hormones.

### Oxidative stress indicators

**Fig 5:**
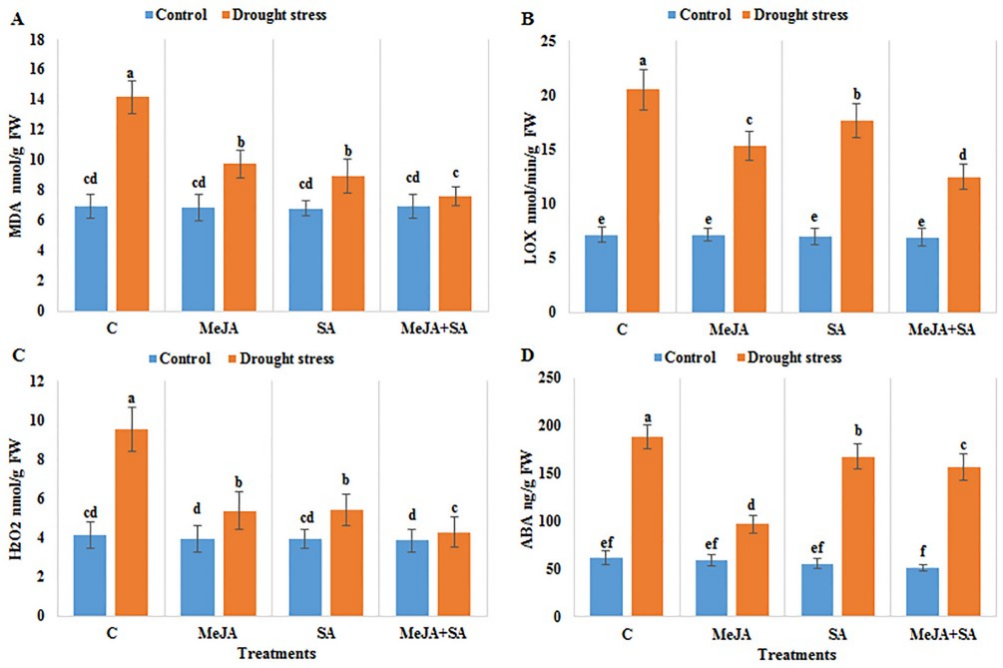
Effect of exogenously applied MeJA and SA on (A) MDA (B) LOX activity (C) H_2_O_2_ and (D) ABA content of maize seedlings grown under control (well-watered) and drought stress conditions. The data represent means of three replicates and error bars showing ± standard errors of the mean values. Mean values that share the same letter are not-significantly different; otherwise they differ at p<0.05.

Oxidative stress due to drought treatment in maize seedlings was determined via assay of lipid peroxidation and production of reactive oxygen species (ROS). Indicators of oxidative stress, namely malondialdehyde (MDA), lipoxygenase (LOX) activity and hydrogen peroxide (H_2_O_2_), were increased in drought-stressed plants compared to the well-watered controls (Fig 5A, 5B and 5C). Drought stress increased the MDA, LOX activity and H_2_O_2_ by 104, 188 and 131%, respectively, compared to the well-watered controls. The exogenous application of MeJA+SA resulted in a lower MDA, LOX activity and H_2_O_2_ concentrations by 46, 39 and 55%, respectively, in maize plants under drought stress compared to drought-stressed plants without pre-treatment with exogenous hormones.

### Effects of MeJA and SA on endogenous ABA under drought stress

Drought stress exhibited a remarkable increase in ABA content by 127% compared to the well-watered controls (Fig 5D). The exogenous applications of MeJA, SA and MeJA+SA alleviated the drought stress-induced increase in ABA content compared to un-treated stressed plants. MeJA alone was mitigated the sharp rise in ABA by 49% most effectively compared to drought-stressed plants not pre-treated with exogenous hormones.

### Effects of MeJA and SA on antioxidant enzyme activities

**Fig 6:**
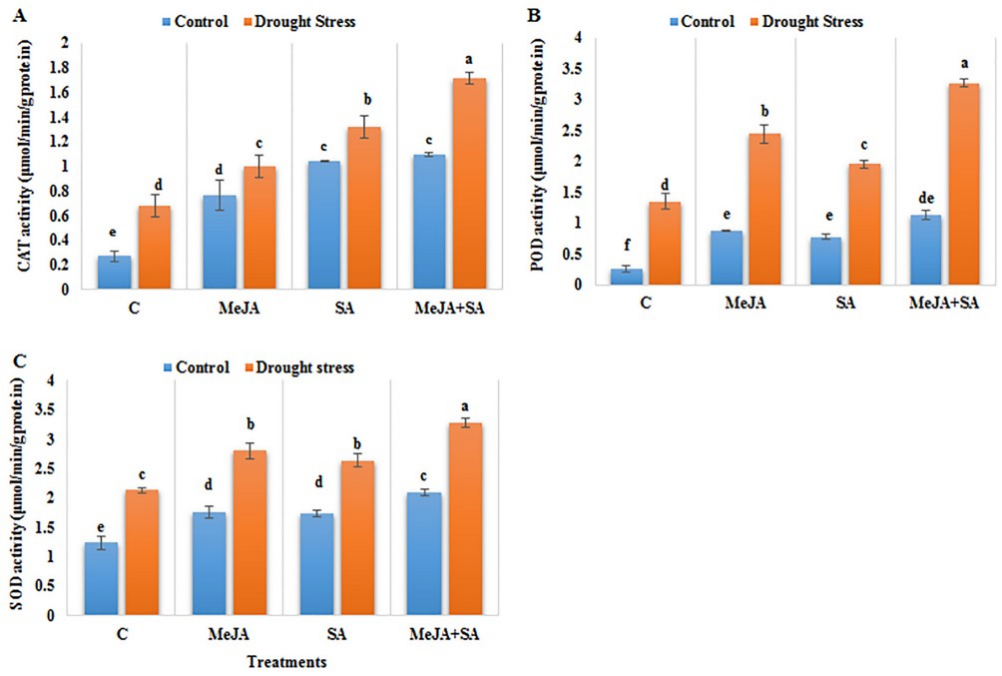
Effect of exogenously applied MeJA and SA on (A) catalase activity (B) peroxidase activity and (C) superoxide dismutase activity of maize seedlings grown under control (well-watered) and drought stress conditions. The data represent means of three replicates and error bars showing ± standard errors of the mean values. Mean values that share the same letter are not-significantly different; otherwise they differ at p<0.05.

Drought stress led to significant increases by 151, 435, and 73% in CAT, POD and SOD activity, respectively, in maize plants as compared to the well-watered control plants (Fig 6A, 6B and 6C). Exogenous pre-treatments with MeJA, SA and MeJA+SA to drought-stressed plants further increased the catalase (CAT), peroxidase (POD) and superoxide dismutase (SOD) activities compared to drought-stressed plants not pre-treated with exogenous hormones. The maximum increases in CAT, POD and SOD activity were observed in the MeJA+SA treated maize plants under drought stress, which were 152, 143 and 54%, respectively.

### Heatmap analysis for stress indicators, osmolytes and antioxidant enzyme activities from maize seedlings

**Fig 7:**
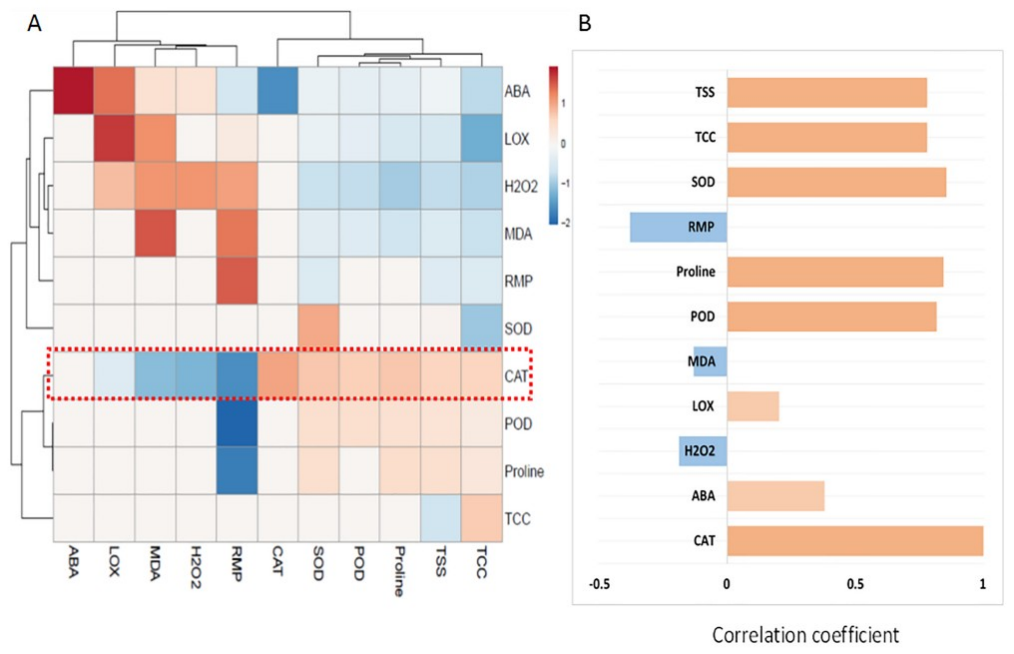
Heatmap analysis of Pearson’s correlation for the targeted stress indicators, osmolytes and antioxidant enzymes in maize seedlings (A and B) under control and drought stress conditions. Pink and blue color indicates positive and negative correlations, respectively. The studied factors were correlated with catalase activity (CAT).

The data on stress indicators, osmolytes and antioxidant enzymes for the maize seedlings treated with MeJA and SA under drought stress were analysed using a heatmap. A positive correlation was found between catalase activity and osmolytes as well as with ROS-related components and stress indicators (Fig 7A). Comparative analysis of these factors related to catalase activity (highlighted by a red box in Fig 7A) suggested that there was a positive correlation with the ABA, LOX, POD, proline, SOD, TCC and TSS while a negative correlation with H_2_O_2_, MDA and RMP (Fig 6B).

### Principal Component Analysis (PCA) for the targeted stress indicators, osmolytes and antioxidant enzyme activities from maize seedlings

**Fig 8:**
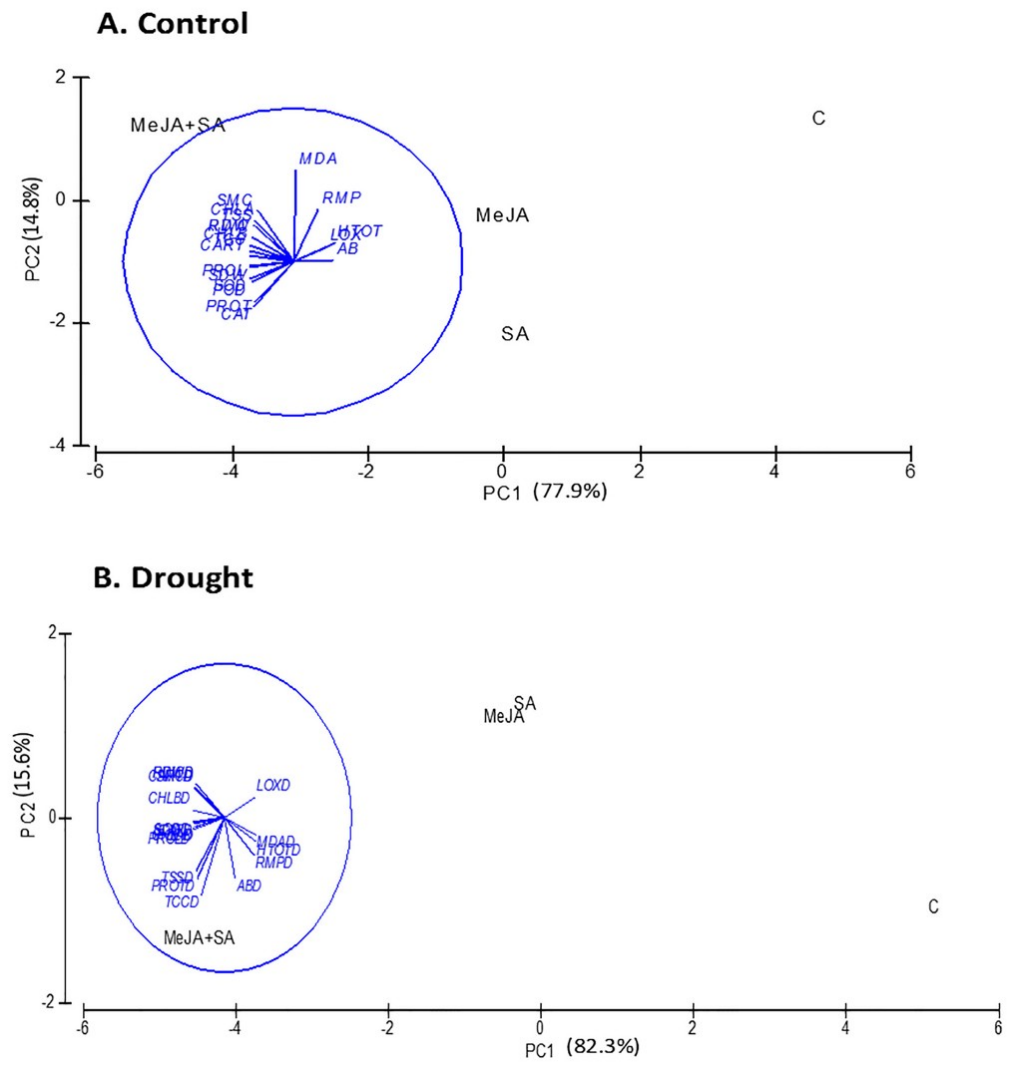
Principal component analysis (PCA) for the targeted stress indicators, osmolytes and antioxidant enzymes from maize seedlings under (A) control and (B) drought stress conditions.

The responses of maize seedlings to MeJA and SA applications under well-watered (control) and drought stress conditions were evaluated via a PCA score plot (Fig 8A and 8B). The PCA plot for the well-watered plants accounted for 77.9% and 14.8% of the variance of PC1 and PC2, respectively, while the plot for the drought stressed plants gave corresponding values of 82.3% and 15.6%. The PCA results showed that maize plants exhibited a similar pattern of plant physiological and biochemical responses according to the MeJA+SA application under control and drought stress. These results suggest that MeJA+SA application is more effective under stress conditions in increasing the osmolyte concentrations for the osmotic adjustment and antioxidant enzymes for the alleviation of oxidative stress caused by drought stress (Fig 8B).

## Discussion

In this study, the potential of exogenous application of two growth regulators, methyl jasmonate (MeJA) and salicylic acid (SA), was evaluated for its ability to improve the morpho-physiological growth and biochemical attributes of maize under drought stress. Maize growth (root and shoot lengths; fresh and dry weights) was reduced due to the stress while the MeJA and SA applications mitigated the stress-imposed adverse effects. Drought stress is known to cause drastic reductions in maize plant growth and to disrupt developmental processes, leaf area, photosynthetic rate, metabolic activities, with reduction in its biomass being one of the main drought stress indicators [6,7,54]. For example, Anjum et al. [55] showed in soybean that water stress significantly reduced the number of nodes as well as plant height, stem diameter, number of branches and leaf area.

Jasmonic acid (JA) is known to play a positive role in promoting plant primary root growth and cell elongation, cell expansion and cell differentiation of potato stolons [56,57]. Tavallali and Karimi [58] reported significantly higher plant root growth in MeJA-treated almond plants under salt stress conditions, which could be due to MeJA translocation to roots and induction of defense/resistance responses in the host plant. Soaking maize seeds with MeJA alleviated the detrimental effects of drought stress by increasing plant height, fresh and dry weights, chlorophyll pigments and antioxidant activities [14]. Exogenous application of SA and MeJA to plants under drought stress conditions induces various biochemical, morphological and physiological responses [55] and the same treatments enhanced drought stress tolerance in chamomile plants [59]. It has been shown that wheat plant treatment with SA may confer resistance to drought by increasing the cell division of plant roots within the apical meristem [60,61].

The relative water content (RWC), proline, relative membrane permeability (RMP), total soluble sugars (TSS) and total carbohydrate content (TCC) of leaves from the drought-treated and control maize plants were evaluated. Inhibition of electron transport, as well as reduction in RWC and chlorophyll content have been identified as plant stress indicators [62,63]. MeJA+SA increased leaf RWC, protein, chlorophyll and carotenoid contents of maize plants under water stress. The same treatment significantly decreased membrane damage as reflecting in a decrease in RMP compared to the drought-treated plants without pre-treatment with plant hormones. The results are in accordance with Abdelgawad et al. [23] and Chen et al. [64] who reported the protective potential of MeJA in improving maize and *Kandelia obovate* plant growth, yield and enhancing drought tolerance.

A positive correlation between RMP and sensitivity to drought stress has been reported [65]. Drought causes an imbalance in plant water status, disrupts osmotic adjustment and ultimately leads to accumulation of compatible osmolytes in plants [66]. Reduced RWC was observed along with the accumulation of compatible compounds like proline, TSS and TCC. Exogenous applications of MeJA+SA resulted in increased RWC, proline, TSS and TCC under stress conditions, suggesting a role in the restoration of plant tissue water and thus osmoprotection. Osmoprotectants are known to increase the osmotic pressure in cytoplasm, for the maintenance of turgor pressure and water uptake (osmotic adjustment) [67]. Other than osmotic adjustment, these organic compounds are also reported to play their role as scavengers of reactive oxygen species (ROS), and help in metabolic detoxification [68].

The exogenous applications of MeJA and SA more significantly increased the proline content compared to control under drought stress [69]. Reduction in the volume of chloroplast stroma and production of reactive oxygen species (ROS) are involved in the inhibition of photosynthesis during water stress [70]. Singh and Usha [71] reported that SA application increases stomatal conductance and chlorophyll content. Drought stress is associated with the depletion of starch in plant cells, as starch breakdown results in the accumulation of soluble sugars under stress conditions [72]. Hoekstra and Buitink [73] reported a very strong correlation between the levels of plant soluble sugars and drought tolerance. Anjum et al. [74] stated that MeJA application helped plants to maintain RWC and further improved the proline content of soybean plants under stress conditions. The same treatments significantly enhanced the tolerance of *Matricaria chamomilla* against drought stress while increasing the levels of soluble sugars and proline [59].

The effect of exogenous hormone pre-treatments on oxidative stress during water deficit was investigated by determining concentrations of malondialdehyde (MDA) and hydrogen peroxide (H_2_O_2_). MDA is produced under stress conditions by lipid peroxidation; thus, higher levels of MDA are positively correlated with the extent of membrane damage. Furthermore, ROS such as hydroxyl radicals (OH), superoxide radical (O_2_^−^) and H_2_O_2_, cause lipid peroxidation of plant membranes under abiotic stresses [75]. The results showed that drought stress resulted in increased levels of H_2_O_2_, MDA and endogenous phytohormone ABA compared to healthy control plants. Exogenous applications of MeJA+SA significantly reduced H_2_O_2_ and MDA content.

Increased levels of endogenous ABA content in the MeJA and MeJA+SA treated plants were observed compared to the stress control. ABA is known to confer tolerance/resistance in plants to water deficit [76]. Increased endogenous ABA enhanced tolerance in rice (*Oryza sativa*) and soybean (*Glycine max*) to stress conditions [77].

MeJA+SA treatment (10 μM + 1 mM) enhanced the antioxidant defense enzyme activities of the maize leaves including CAT, POD and SOD under drought stress and mitigated the oxidative damage instigated by drought. These results are in accordance with Majid and Akbar [78], who observed the positive role of MeJA in increasing antioxidant enzyme production under PQ-induced oxidative stress. The exogenous application of MeJA and JA to plants under abiotic stresses also improves the activities of antioxidants and helps in the neutralization of ROS and, provides protection against oxidative stress [78,79]. The CAT, POX and SOD antioxidant enzyme activities were increased in the MeJA- and SA-treated plants (separately) under drought stress conditions [80,23]. However, MeJA (20 μM) and SA (2 mM) applied alone did not significantly improve these activities under non-stress control conditions because the effectiveness of MeJA or SA is generally dependent on other stress signals for their endogenous activation and upregulation of antioxidant enzyme activities [57]. Other studies have found that SA at high concentrations (2–3 mM) is toxic, suppressing plant growth and drought tolerance in wheat seedlings [81] and may promote the formation of ROS in photosynthetic tissues and increase oxidative damage during osmotic stresses [82]. SA application ranging from 0.1–1 mM in muskmelon seedlings improved drought tolerance [83]. However, despite this general effect, the results on maize indicate that 2 mM promoted growth to some extent compared to control plants, but it was more effective in increasing maize plant growth and drought tolerance at 1 mM concentration in combination with 10 μM MeJA.

## Conclusion

The exogenous applications of MeJA (20 μM) and SA (2 mM) alone reduced the harmful effects of drought stress in maize to some extent compared to the untreated control. MeJA applications resulted in a maximum increase in endogenous abscisic acid (ABA) under drought stress. However, the combined half-dose treatments of MeJA+SA (10 μM + 1 mM) more effectively alleviated the adverse effects of drought-induced oxidative stress. The same treatment gave the maximum increase in osmolytes, thereby maintaining the water status of the maize plants, and also increased the antioxidant enzyme activities under drought stress conditions. In conclusion, exogenous applications of MeJA+SA as combined seed and foliar pre-treatments can partially prevent the harmful effects of drought-induced oxidative and osmotic stress in maize seedlings by modulating osmolytes, endogenous hormones and antioxidant enzymes.

## Supporting information

S1 Dataset Pot experiment data. (XLSX)
